# Cinnamon extract induces tumor cell death through inhibition of NFκB and AP1

**DOI:** 10.1186/1471-2407-10-392

**Published:** 2010-07-24

**Authors:** Ho-Keun Kwon, Ji-Sun Hwang, Jae-Seon So, Choong-Gu Lee, Anupama Sahoo, Jae-Ha Ryu, Won Kyung Jeon, Byoung Seob Ko, Chang-Rok Im, Sung Haeng Lee, Zee Yong Park, Sin-Hyeog Im

**Affiliations:** 1 School of Life Sciences and Immune Synapse Research Center, Gwangju Institute of Science and Technology (GIST), 1 Oryong-dong, Puk-ku, Gwangju 500-712, Republic of Korea; 2 Korea Institute of Oriental Medicine, Daejeon 305-811, Republic of Korea; 3 Global leader program, Bugil Academy, Cheonan, Gyeonggido 330-941, Republic of Korea; 4 Chosun University School of Medicine, Gwangju 501-759, Republic of Korea

## Abstract

**Background:**

*Cinnamomum cassia *bark is the outer skin of an evergreen tall tree belonging to the family Lauraceae containing several active components such as essential oils (cinnamic aldehyde and cinnamyl aldehyde), tannin, mucus and carbohydrate. They have various biological functions including anti-oxidant, anti-microbial, anti-inflammation, anti-diabetic and anti-tumor activity. Previously, we have reported that anti-cancer effect of cinnamon extracts is associated with modulation of angiogenesis and effector function of CD8^+ ^T cells. In this study, we further identified that anti-tumor effect of cinnamon extracts is also link with enhanced pro-apoptotic activity by inhibiting the activities NFκB and AP1 in mouse melanoma model.

**Methods:**

Water soluble cinnamon extract was obtained and quality of cinnamon extract was evaluated by HPLC (High Performance Liquid Chromatography) analysis. In this study, we tested anti-tumor activity and elucidated action mechanism of cinnamon extract using various types of tumor cell lines including lymphoma, melanoma, cervix cancer and colorectal cancer *in vitro *and *in vivo *mouse melanoma model.

**Results:**

Cinnamon extract strongly inhibited tumor cell proliferation *in vitro *and induced active cell death of tumor cells by up-regulating pro-apoptotic molecules while inhibiting NFκB and AP1 activity and their target genes such as *Bcl-2*, *BcL-xL *and *survivin*. Oral administration of cinnamon extract in melanoma transplantation model significantly inhibited tumor growth with the same mechanism of action observed *in vitro*.

**Conclusion:**

Our study suggests that anti-tumor effect of cinnamon extracts is directly linked with enhanced pro-apoptotic activity and inhibition of NFκB and AP1 activities and their target genes *in vitro *and *in vivo *mouse melanoma model. Hence, further elucidation of active components of cinnamon extract could lead to development of potent anti-tumor agent or complementary and alternative medicine for the treatment of diverse cancers.

## Background

Herbal medicines are plant-derived products which have been used as traditional folk medicine and food additives. Recently their medicinal properties are under extensive investigation and become a major part of complementary and alternative medicines (CAMs). Their potency for treating different diseases has been reported including cancer, allergy and diabetes [1-4].

*Cinnamomum cassia *bark is the outer skin of an evergreen tall tree belonging to the family Lauraceae. Its extracts contain several active components such as essential oils (cinnamic aldehyde and cinnamyl aldehyde), tannin, mucus and carbohydrates [5,6]. They have various biological functions including anti-oxidant, anti-microbial, anti-inflammation, anti-diabetic effects [7-12], and anti-tumor activity [11,13]. However, for the development of cinnamon as CAMs for cancer treatment, further studies are necessary such as elucidation of working mechanisms and characterization of active compounds directly linked with anti-tumor activity.

Cancers are the most life-threatening health problems in the world [14]. There have been many trials to treat cancers through modulation of anti-tumor immune response, apoptosis and anti-tumor proteins [15-18]. Tumor cells are generally resistant to apoptosis; hence selective killing of tumor cells by promoting apoptosis pathway is an attractive and effective way for development of anti-cancer agents. NFκB and AP1 constitutively active in many kinds of cancers and play critical roles in tumor development and progression through modulation of their target genes involved in angiogenesis, metastasis and cell survival [19-21].

Recently we have reported that anti-cancer effect of cinnamon extracts is associated with modulation of angiogenesis and effector function of CD8^+ ^T cells [22]. In this study we further identified that anti-tumor effect of cinnamon extracts is also linked with their enhanced pro-apoptotic activity by inhibiting the activities of NFκB and AP1 in mouse melanoma model.

## Methods

### Animals

C57BL/6 mice (6~8 weeks, male) were purchased from SLC (Japan) and maintained under specific pathogen-free conditions in an animal facility at the Gwangju Institute of Science and Technology (GIST). All of the animal experiments were approved by the GIST Animal Care and Use Committee.

### Preparation of cinnamon extract

Dried *Cinnamomum cassia *bark (Hwajin Distribution Co., Seoul, Korea) was pulverized and extracted for three hours in a hot water extractor. The extract was filtered and the supernatant was concentrated with a rotary evaporator. The extract was then freeze dried resulting in a powder extract. The powder extract was suspended in sterilized distilled water at appropriate concentrations. As we reported in our previous work [22], HPLC analysis was performed by comparing the levels of *trans*-cinnamic acid (Sigma, USA) and cinnamic aldehyde (kindly provided by Dr. Ehren., Germany) as known standards makers for the quality control of composition of cinnamon extract in each experiment. Chromatography was carried out using 1% acetic acid (H_2_0)-MeOH (50: 50 v/v) at room temperature on a Phenomenex Luna 5u C_18_, 100 A pore size, 250 × 4.60 mm I.D. column. The flow rate of the mobile phase was 2 ml/min. The amount of *trans*-cinnamic acid and cinnamic aldehyde was about 2.9 (mg/g extract) and 7.9 (mg/g extract) in each extract [22].

### Cell lines

B16F10 and Clone M3 (mouse melanoma cell), Hela (human cervical carcinoma cell) and Caco2 (human epithelial colorectal adenocarcinoma cell) were obtained from the Korean Cell Line Bank (Seoul National University, Korea) and maintained in Dulbecco's modified Eagle's medium (DMEM) supplemented with 10% fetal bovine serum (Hyclone Laboratories, Logan, USA), 100 U/ml penicillin (Sigma) and 100 μg/ml streptomycin (Sigma). To check effects of cinnamon extract in normal cells, primary mouse lymphocytes were isolated and cultured in Dulbecco's modified Eagle's medium (DMEM) supplemented with 10% fetal bovine serum, L-glutamine, penicillin-streptomycin, nonessential amino acids, sodium pyruvate, vitamins, HEPES and 2-mercaptoethanol.

### Cell viability analysis

Cell viability and proliferation were determined with EZ-Cytox Cell Viability Assay Kit (Daeil Labservice, Korea) based on the cleavage of the tetrazolium salt to water-soluble formazan by succinate-tetrazolium reductase. Briefly, cells were treated with cinnamon extract (0.5 mg/ml) or Doxorubicin (Sigma) for indicated time points in 6 well plates. After treatment, cells were transferred into 96 well plates in 100 μl of medium and incubated with 10 μl of Ez-CyTox solution for 5 hours in the 37°C incubator. Then absorbance were measured using the Easy Reader EAR 400 (SLT-Lab Instruments, Austria) at 420~480nm. Data was presented by relative growth inhibition to PBS treated cells.

### Cell cycle analysis

The effect on cell division by cinnamon treatment was determined by assessing cellular DNA content using propidium iodide (PI) staining [23]. Briefly, cells were treated with 0.5 mg/ml of cinnamon extract for indicated time periods and then each sample was harvested and fixed in 70% ethanol for 10 hours. After fixation, cells were washed with PBS, treated with 0.5 μg/ml of DNase-free RNase (Sigma) for 20 mins at room temperature and stained with 100 μg/ml of PI in 0.1 M sodium citrate buffer (pH 7.4) for 30 mins at 4°C. Flow cytometric analysis (FACS) was performed with EPICS XL Cytometer (Beckman Coulter) and cell cycle distribution was determined with Expo32 program (Beckman Coulter)

### Apoptosis analysis

Cells (1 × 10^6^) were treated with cinnamon extract (0.5 mg/ml) for indicated time periods and then resuspended in 1ml of 1× Annexin V binding buffer (BD bioscience). After incubating for 15 mins with 5 μl of Annexin V-PE and 7-ADD, at 25°C in the dark, 400 μl of 1× binding buffer was added to each tube and immediately analyzed by FACS. Cells stained with isotype matched normal IgG used as a control and showed less than 0.2% positive population (data not shown).

### Luciferase assay

B16F10 cells were transfected with AP1- or NFκB-dependent reporter construct that contains repeated copies of NFκB or AP1 response elements. After 18 hours culture in complete media, cells were stimulated with PMA (phorbol 12-myristate 13-acetate) and ionomycin (P+I) for 4 hours in the presence or absence several dose of cinnamon extract from 0.1 mg/ml to 0.5 mg/ml. Luciferase activity measured by dual luciferase assay system (Promega) is expressed relative to expression of the cotransfected Renilla luciferase promoter (phRL-null; Promega) to control for transfection efficiency.

### RNA isolation, cDNA synthesis, quantitative RT-PCR and standard RT-PCR

Total RNA was prepared using TRI Reagent (Molecular Research Center) according to the manufacturer's protocol. For reverse transcription, cDNA was generated using 1 μg of total RNA, oligo (dT) primer (Promega) and Improm-II Reverse Transcriptase (Promega) in a total volume of 20 μl. One μl of cDNA was amplified using the following RT-PCR primer sets: L32 (5'-GAGGACCAAGAAGTTCATCAG-3' and 5'-GCACAGTAAGATTTGTTGCAC-3'), BcL-xL (5'-GACAAGGAGATGCAGGTATTGG-3' and 5'-TCCCGTAGAGATCCACAAAAGT-3'), Bcl-2 (5'-ATGCCTTTGTGGAACTATATGGC-3'); Bak (5'-GTGACCTGCTTTTTGGCTGAT-3' and 5'-GGTCTCTACGCAAATTCAGGG-3'); Bax (5'-TGAAGACAGGGGCCTTTTTG-3' and 5'-AATTCGCCGGAGACACTCG-3'); Bim (5'-CCCGGAGATACGGATTGCAC-3' and 5'-GCCTCGCGGTAATCATTTGC-3'); Bad (5'-AAGTCCGATCCCGGAATCC-3' and 5'-GCTCACTCGGCTCAAACTCT-3') and 5'-GGTATGCACCCAGAGTGATGC-3'), and Survivin (5'-CTACCGAGAACGAGCCTGATT-3' and 5'- AGCCTTCCAATTCCTTAAAGCAG-3').

### Preparation of nuclear extracts

Cell lines or cells isolated from tumor tissues were washed twice with ice cold PBS and incubated in 1ml of lysis buffer (10 mM Tris/HCl, 3 mM CaCl_2_, 2 mM MgCl_2_) containing a protease inhibitor cocktail (Roche) for 10 mins on ice. Then the cells were vortexed gently and incubated in 1ml of NP-40 buffer (10 mM Tris/HCl, 3 mM CaCl_2_, 2 mM MgCl_2_, 1% NP-40) for 5 mins at 4°C, and the suspension was centrifuged at 3000 rpm for 10 mins at 4°C. Nuclei was washed with 1ml of Buffer A (20 mM Hepes-KOH, 1.5 mM MgCl_2_, 10 mM KCl, 0.5 mM DTT, 0.5 mM PMSF), and 100 μl of Buffer C (20 mM Hepes-KOH, 25% Glycerol, 420 mM NaCl, 1.5 mM MgCl_2_, 0.2 mM EDTA, 5 mM DTT, 0.5 mM PMSF, 1% Triton X-100) was added to the pellet and vortexed vigorously at 4°C for 10 mins. Nuclear debris was removed by centrifugation at 13000 g for 5 mins. Protein concentrations were determined by the Bradford Assay (Bio-Rad). The nuclear extract was confirmed by immunoblotting with anti-Lamin B and anti-Tubulin beta. For single cell suspension of tumor tissues, tumor tissues from each group was homogeized with homogenizer (Fluko).

### Immunoblotting

Proteins were resolved by 10% (for NFκB and AP1) or 15% (for caspase-3, Bcl2, Bcl-xL, Bad, Bax, Bak, Bim and Sruvivin) SDS-PAGE gels, transferred onto a PVDF membrane (Bio-RAD) and subjected to Western blot analysis using anti-NFκB (Abcam), anti-pc-JUN (SantaCruz), anti-caspases-3 (Abcam), anti-Bcl-2 (Abcam), anti-Bcl-xL (Cell signaling), anti-Survivin (Cell signaling), anti-Bad (Abcam), anti-Bax (Abcam), anti-Bak (Abcam), anti-Bim (Abcam) and peroxidase-conjugated secondary antibodies (DAKO). Proteins were visualized with a chemiluminescence kit (Amersham Bioscience). The levels of Tubulin (anti-tubulin; Santa Cruz), beta-actin (anti-beta-actin; Abcam) and Lamin B (anti-lamin B; SantaCruz) detected by relevant antibodies were monitored as a loading control.

### Melanoma induction and anti-tumor assay

Mouse melanoma B16F10 (1 × 10^6 ^cells/0.1ml) cells were injected subcutaneously (s.c) into the flanks of C57BL/6 mice (6 weeks old male). One week after the injection, mice were divided into two groups (10 mice/each group) and orally treated with either 10 mg/dose (400 μg/g mouse weight) of cinnamon extract in 100 μl of PBS or same volume of PBS alone as a sham control for 30 days. During the treatment period, the tumor size was measured with vernier calipers every 2 days, and tumor volumes were calculated using the standard formula: width^2 ^× length × 0.52. Mice were sacrificed for further analysis after 30 days of treatment.

### DNA fragmentation assay

Genomic DNA isolation was performed with gDNA purification kit (Solgent, Korea). Briefly, mouse tumor tissues from the differentially treated group were collected, pooled, and 5 mg of tumor tissues from each group were transferred. They were dissolved in 300ml of cell lysis buffer with 25 mg of proteinase K for 4 hours at 55°C, and then mixed with 100ml of protein precipitation solution. Then solution was centrifugated at 14000 rpm for 3 mins. After centrifugation, DNAs was precipitated, washed with isopropanol and 70% ethanol. DNA pellets were dissolved in 100 μl of DNA hydration solution. Finally, fragmented DNAs (10 ml) were visualized in 2% agarose gels.

### Nuclear staining

Cells seeded on the glass in 12 well plate were incubated with cinnamon extract for 72 hours, washed with PBS and fixed with 4% paraformaldehyde for 15 mins at RT. Fixed cells were incubated in PBS (pH 7.4) containing 200mg of DNase-free RNase (Sigma) for 30 mins at 37°C and stained with 2 mg/ml of Hoechst for 10 mins at 37°C. Nuclear morphology of the cells was observed under fluorescence microscope.

### Statistical analysis

A two-tailed Student's *t*-test was employed where P < 0.05 was considered to be statistically significant (*p < 0.05, **p < 0.005, and ***p < 0.001).

## Results

### Cinnamon extract inhibits tumor cell growth *in vitro*

**Figure 1:**
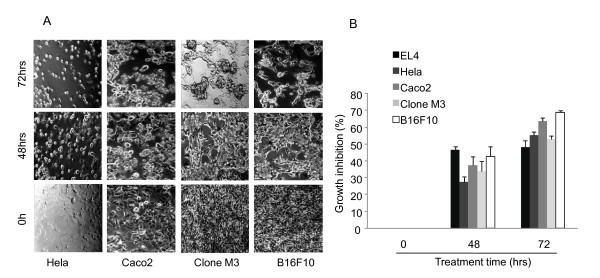
**Treatment of cinnamon extract inhibits the growth of various cancer cells**. Cinnamon (0.5 mg/ml) was treated for 0, 48 and 72 hrs to cancer cell lines (Hela; cervical cancer, Caco2; colon cancer, Clone M3; melanoma and B16F10; melanoma). After treatment of cinnamon extract, (A) morphological changes of each cancer cell lines were monitored by microscopic observation. (B) Proliferation and viability of cancer cells were measured by cell viability assay at the indicated time points. Error bars indicated SD. Data are representative of three independent experiments.

To define the optimal concentration at which cinnamon extract does not induce cell damage, the cytotoxicity test was performed in melanoma cell lines. As we reported previously, treatment of cinnamon extract up to 0.5 mg/ml did not induce growth inhibition and morphological changes till 24 hrs [22]. However, tumor cells showed significant decrease in cell proliferation after 48 hrs treatment of cinnamon extract (Figure 1). They showed condensed, shrank and aggregated shapes. Seventy two hours after treatment of cinnamon extract, most of the cells became floating with aggregated form detached from the plates (Figure 1A). To confirm the effects of cinnamon treatment on tumor cell survival, proliferation and cell viability were measured (Figure 1B). In the presence of cinnamon extract, the rate of tumor cell growth was significantly inhibited (Figure 1B). To validate our experimental system, we used EL4 T lymphoma cell line as a positive control that showed cell cycle arrest and growth inhibition upon treatment of polyphenolic compound from cinnamon [24]. We checked whether cinnamon extract also induced growth inhibition of normal cells. Primary mouse lymphocytes were treated with same concentration (0.5 mg/ml) of cinnamon extracts and cell viability was measured (Additional file 1, Figure S1A). Interestingly, treatment of cinnamon extract (0.5 mg/ml) did not induce any growth inhibition in primary mouse lymphocyte.

### Treatment of cinnamon extract induces active cell death of melanoma cells *in vitro*

**Figure 2:**
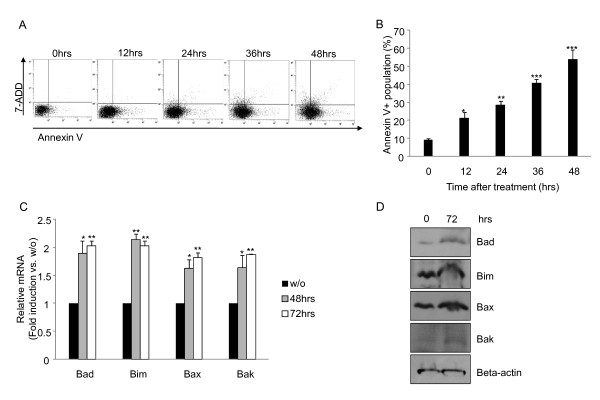
**Cinnamon extract treatment induces active cell death in melanoma cells**. At the indicated time periods after treatment of cinnamon extract, cells were stained with Annexin V and 7-ADD. Double positive (Annexin V^+ ^and 7-ADD^+^) (A) or Annexin V^+ ^positive (B) populations were analyzed by FACS. Gene expression (C) and protein (D) level of pro-apoptotic molecules such as Bad, Bim, Bax and Bak were measured by quantitative real-time PCR or immunoblotting, respectively. Error bars indicated SD. One (*), two (**) or three asterisks (***) indicate p < 0.05, p < 0.005 or p < 0.001, respectively. Data are representative of three independent experiments.

Growth inhibition of tumor cells by the treatment of cinnamon extract could be mediated by several mechanisms. A recent report showed that phenolic compound of cinnamon bark induces cell cycle arrest in hematologic cell lines [11]. Hence, we first tested whether cinnamon extract induced cell cycle arrest in mouse melanoma cell line, B16F10 cells. Treatment of cinnamon extract slightly induced cell cycle arrest at S phage (Additional file 2, Table S1). We further tested whether growth inhibition by treatment of cinnamon is related with induction of apoptosis (Figure 2). Although cinnamon treatment did not induce growth inhibition and morphological chance within 24 hrs [22], a gradual increase in early apoptotic population (Annexin V^+^) was observed (Figure 2A and 2B; 12 and 24 hrs). Further enhancement of apoptotic population in late stage (Annexin V^+^/7-ADD^+^) was observed in a time-dependent manner (Figure 2A). To further confirm these results, we checked whether treatment of cinnamon extract induces apoptosis in another type of cancer cell line, Caco2 (human epithelial colorectal adenocarcinoma cell). Like in B16F10 melanoma cells, treatment of cinnamon extract induced apoptosis in Caco2 as well (Additional file 3, Figure S2). From these experiments, we could infer that cinnamon extract induced apoptosis in cancer cells rather than showing simple cytotoxic effects. Next, we tested whether cinnamon extract induced apoptosis is affected by changes in the expression level of apoptosis related genes [25]. Indeed, treatment of cinnamon extract significantly increased mRNA expression (Figure 2C) and protein (Figure 2D) levels of pro-apoptotic genes such as Bad, Bim, Bax and Bak.

### Cinnamon extract inhibits the melanoma growth by inhibiting NFκB and AP1

**Figure 3:**
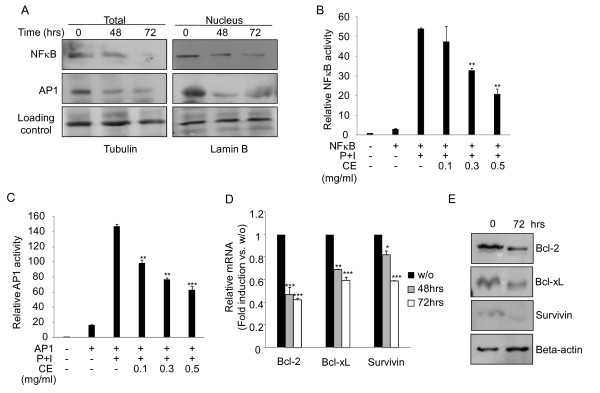
**Cinnamon extract treatment down-regulates the levels of NFκB and AP1 and their target genes**. (A) After treatment of cinnamon extract for indicated time periods, the levels of NFκB and AP1 in total cell lysates (left) and nucleus extracts (right) were compared by immunoblotting. The effect of cinnamon extract on the activity NFκB (B) and AP1 (C) was measured by reporter assay. B16F10 cells were transfected with AP1- or NFκB-dependent reporter construct and then stimulated with PMA and ionomycin (P+I) for 4 hours in the absence or presence of cinnamon extract (CE). Gene expression (D) and protein (E) levels of anti-apoptotic genes were measured by quantitative real-time PCR or immunoblotting, respectively. Data are representative of three independent experiments with similar results. One (*), two (**) or three asterisks (***) indicate p < 0.05, p < 0.005 or p < 0.001, respectively.

NFκB and AP1 have critical roles in tumor cell survival. They regulate the gene expression program of apoptosis and cell cycle [21,26]. Hence, we checked whether a pro-apoptotic activity of cinnamon extract is linked with changes in the levels and activities of NFκB and AP1. Interestingly, cinnamon extract decreased the amount of NFκB and AP1 proteins in total cell lysates (left panel in Figure 3A) as well as in nucleus extracts (right panel in Figure 3A). To further test whether cinnamon extract also affected on NFκB and AP1 activities, luciferase-based reporter assay was performed. B16F10 cells were stimulated with PMA + ionomycin (P+I) to activate NFκB and AP1 in the absence or presence of cinnamon extract. Indeed, treatments of cinnamon extract (CE) significantly down-regulated NFκB and AP1 activities in a dose dependent manner (Figure 3B and 3C). Next, we checked whether down-regulation of NFκB and AP1 levels and their activities by cinnamon treatment could also affect expression level of their target genes related with apoptosis and cell survival such as Bcl-2, BcL-xL and survivin [21,27]. Indeed, cinnamon extract significantly down-regulated the expression (Figure 3D) and protein (Figure 3E) levels of Bcl-2, BcL-xL and survivin in a time dependent manner. These results suggest that reduction in the levels and activities of NFκB and AP1 by cinnamon extract down-regulated their target molecules involved in tumor cell survival.

### Oral administration of cinnamon extract significantly inhibits melanoma progression *in vivo*

**Figure 4:**
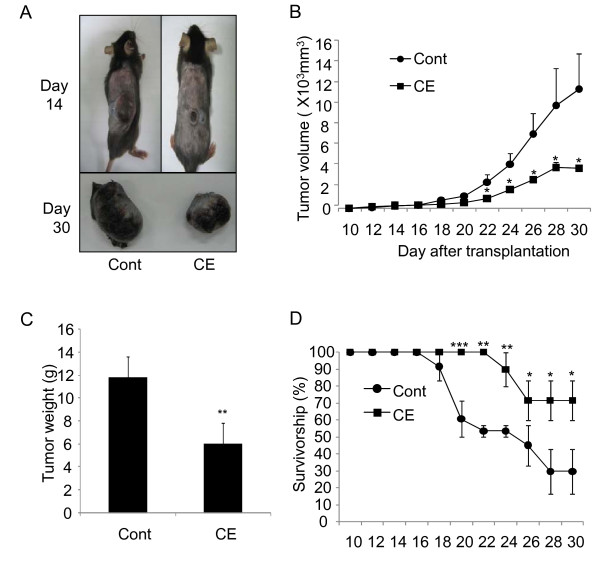
**Oral administration of cinnamon extract inhibits melanoma growth *in vivo***. Ten days after melanoma transplantation, cinnamon extract (CE; 400 μg/g mouse weight) or PBS (Cont) was orally administrated every two days for 20 days. (A) Photographs of representative tumors from each group at day 14 and 30. During the treatment period, the tumor volumes (B) and tumors weights (C) and survivor rate (D) in each group were daily measured. Data are representative of three independent experiments (10 mice/each group). One (*) or two (**) asterisks indicates p < 0.05 or p < 0.005, respectively. Data are representative of three individual experiments.

To further confirm the anti-tumor effect of cinnamon extract *in vivo*, we orally administrated cinnamon extract to a mouse melanoma model. Ten days after subcutaneous transplantation of melanoma cells (B16F10), mice were divided into two groups. Mice in each group were daily treated with cinnamon extract (CE; 400 μg/g mouse weight) or same volume of PBS as a sham control group (Cont) for 30 days by oral-administration with catheter (Figure 4). The dose of cinnamon extract in oral administration was based on our previous work [22] that did show any cytotoxic effect in normal mice. To test anti-tumor effects of cinnamon extract, tumor volume was measured throughout the treatment period (Figure 4B). Oral administration of cinnamon extract significantly reduced tumor size compared with control group (Figure 4A and 4B). Consistent with tumor volume, cinnamon treated group showed significant decrease of tumor weight compared with control groups (cinnamon group; 6.2 g vs. control group; 12.1 g) (Figure 4C). A reduction in tumor size by administration of cinnamon extract significantly increased a survivor rate compared with control group (Figure 4D). These data suggests that oral administration of cinnamon extract has potent anti-tumor activity *in vivo*.

### Anti-tumor effect of cinnamon extract is linked to the reduced levels of NFκB and AP1 *in vivo *melanoma model

**Figure 5:**
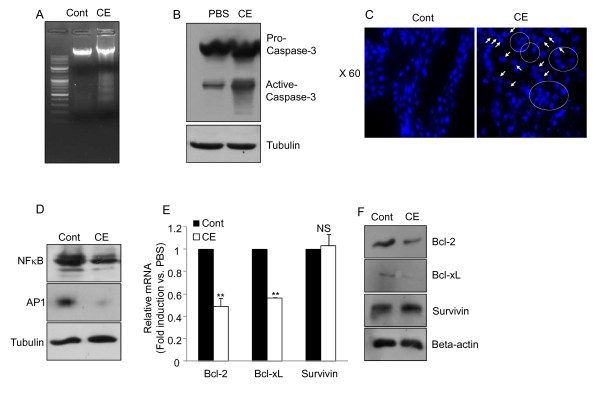
**Cinnamon extract treatment induces tumor apoptosis by decreasing the levels of NFκB and AP1 and their target genes**. (A) Genomic DNA was isolated from tumor tissues from each treatment group and DNA fragmentation was confirmed by staining with ethidium bromide in 2% agarose gel. (B) The protein levels of pro-caspase and active caspases 3 in tumor tissues from each group were determined by immunoblotting. (C) To check the structural changes of nucleus, tumor tissues from each group were sectioned and then stained with Hoechst. Round areas and arrows indicate apoptotic cells (bigger size of this picture was included in Additional file 4, Figure S3). (D) The levels of NFκB and AP1 in tumor tissues from each group were compared by immunoblotting between the treatment groups. The gene expression (E) and protein (F) level of Bcl-2, BcL-xL and survivin in tumor tissues were measured by quantitative real-time PCR or immunoblotting, respectively. Two (**) asterisk indicates p < 0.005. Not significant (NS). Data are representative of three individual experiments with similar results.

To get the detail action mechanisms of cinnamon *in vivo*, we tried to investigate whether anti-tumoral effects of cinnamon *in vivo *melanoma model is also mediated by the similar mechanisms of *in vitro *system such as active tumor cell death and inhibition of NFκB and AP1 activities (Figure 1 and Figure 2). Apoptosis assay was performed with the cells obtained from the tumor tissues of each treatment group, cinnamon extract (CE) and PBS (cont). First, DNA fragmentation assay was performed [28]. Genomic DNAs purified from each treatment group were separated by gel electrophoresis to compare DNA laddering generated by apoptosis. As shown in Figure 5A, DNA isolated from cinnamon extract treated mice (CE) showed a significant increase in DNA fragmentation levels compared with PBS group (Cont) (Figure 5A). Next, we checked the protein levels of caspase-3. Cinnamon extract treatment did not alter the levels of inactive pro-caspase 3 (Figure 5B). Interestingly, however, cinnamon extract treatment significantly increased the level of active caspases-3 compared with the control group (Figure 5B). In addition, the pro-apoptotic effect of cinnamon extract in tumor tissue was further confirmed by tissue staining with Hoechst dye under microscopic observation. Compared with the PBS treated group, tumor tissue sections isolated from cinnamon treated group (CE) showed enhancement of apoptotic population showing condensed chromatin at the nuclear membrane (crescent formation), dissolution of the nuclear membrane, and apoptotic nucleus surrounded by a rim of cytoplasm and plasma membrane [29] (Figure 5C and Additional file 4, Figure S3). In agreement with *in vitro *data, these data suggest that *in vivo *anti-tumoral effect of cinnamon extract is also linked with significant increment of apoptosis in the melanoma tissues. Next, we examined whether *in vivo *anti-tumoral effect of cinnamon extract is also directly associated with the down-regulation of NFκB and AP1 levels. Indeed, tumor tissues isolated from cinnamon extract treated group (CE) showed a significant reduction in NFκB and AP1 levels compared with PBS treated control group (Cont) (Figure 5D). We also tested whether down-regulation NFκB and AP1 leads to a decrease in the levels of their target genes such as Bcl-2, BcL-xL and survivin. The mRNA expression (Figure 5E) and protein (Figure 5F) levels of these target molecules were analyzed from the tumor tissues of each treatment groups. Indeed, tumor tissues from cinnamon extract treated mice showed a significant decrease in the levels of *Bcl-2 *and *BcL-xL *compared with control group (Figure 5E and 5F). These data indicate that anti-tumoral effects of cinnamon extract is mediated by induction of tumor apoptosis through the inhibition of NFκB and AP1 levels.

## Discussion

Cinnamon is a herbal plant that has been used for various purposes as forms of dietary intake, oriental medicine and CAMs [30]. However, it is still unclear about the exact action mechanisms of cinnamon and its active components related with diverse biological function. Although various beneficial effects of cinnamon extract have been reported, most studies were performed *in vitro *culture system without elucidation of mechanism of action *in vivo*.

In our previous work [22], we have shown that anti-tumoral effects of cinnamon extract in mouse melanoma is mediated by modulation of angiogenesis and cytotoxic activity of CD8^+ ^T cells. In the present study, we further demonstrated that anti-tumoral effects of cinnamon extract are also linked with the induction of apoptosis in a cancer specific manner. In addition, treatment of cinnamon extract reduced the levels and activities of NFκB and AP1 and their target genes such as Bcl-2 and Bcl-xL. These findings strongly suggest that potent anti-tumoral effects of cinnamon extract are mediated by multiple action mechanisms.

Active induction of apoptosis in a cancer specific manner is an attractive way to cure many types of cancers [31,32]. Cancers have various strategies to escape from the recognition and elimination by the surveillance of host immune system. These include altered expression of genes and proteins involved in cell survival, death and transformation [33]. Among them, one of common survival strategy of cancer cells is to escape from apoptosis by deregulation of apoptotic genes [32] or hyper-activation of anti-apoptotic genes [34]. Therefore, cancer specific induction of apoptosis is thought to be a good strategy for cancer treatment. In this study, we demonstrated that treatment of cinnamon extract suppressed melanoma progression *in vivo *(Figure 4 and 5) and inhibition of tumor cell growth *in vitro *(Figure 1 and Figure 2) through apoptosis induction. Compared with known anti-cancer drugs (for example, Doxorubicin) [35], potential benefit of cinnamon extract as a complementary and alternative medicine may contribute to its less cytotoxicity in normal cells (Additional file 1, Figure S1B). To compare cytotoxicity of cinnamon extract with anti-cancer drug (e.g, Doxorubicin), firstly we titrated and decided an optimal concentration of cinnamon extract (CE; 0.5 mg/ml) and Doxorubicin (Dox; 5 μM) [35,36] that does not induce apoptosis in normal cells. Cinnamon extract and Doxorubicin induced comparable level of apoptosis induction in melanoma cells (CE; 60% and Dox; 70%, respectively) (Additional file 1, Figure S1B). Interestingly, however, compared with cinnamon extract, Doxorubicin showed much higher toxic effect in normal cells (primary mouse lymphocyte) (Additional file 1, Figure S1B). Doxorubicin treatment induced significantly higher levels apoptosis (up to 50%) of normal lymphocyte while cinnamon extract induced marginal effect (about 10%) (Additional file 1, Figure S1B). These results suggest a beneficial effect of cinnamon extract with less cytotoxicity than conventional anti-cancer drug in normal cells while maintains its anti-tumor effect. However, further studies are needed to elucidate mechanism of action and core active compounds of cinnamon extract to induce cancer cell apoptosis without affecting normal cells.

NFκB and AP1 play pivotal roles in tumorigenesis [20,21,26]. Interestingly, treatment of cinnamon extract strongly down-regulated the levels and activities of NFκB and AP1 both in melanoma cell line (Figure 3) and in mouse melanoma (Figure 5). NFκB is a major regulator of cell proliferation and cell survival. It inhibits apoptosis while stimulating cell proliferation, metastasis, angiogenesis and inflammation [26]. Anti-apoptotic activities of NFκB is generally mediated by activation of set of genes related with cell survival [27]. Together with NFκB, AP1 has also critical roles in tumorigenesis. It stimulates the expression of anti-apoptotic genes, invasive tumor growth, metastasis and angiogenesis [21]. Bcl-2, BcL-xL and survivin are key anti-apoptotic conductors and are target genes of NFκB and AP1 [25]. Treatment of cinnamon extract significantly down-regulated their mRNA expression and protein levels in tumor cell line (Figure 3D and 3E) and melanoma tissue (Figure 5E and 5F) as well. These results suggest that anti-tumor effect of cinnamon extract is linked with the inhibition of NFκB and AP1 and their target genes involved in tumor cell survival and proliferation. In this study, we demonstrated the anti-tumor effect of cinnamon extract *in vivo *melanoma model. Although cinnamon extracts increased apoptosis in various cancer cell lines such as lymphoma, cervical cancer and colorectal cancer (Figure 1), *in vivo *animal studies are necessary to test whether cinnamon extracts have also anti-tumor effects in other types of cancers. In summary, anti-tumor effects of cinnamon extract appear to be mediated by multiple mechanisms. These include inhibition of angiogenesis, potentiating CD8^+ ^T cell cytotoxicity [22] and apoptosis induction in tumor cells. Collectively, our work suggests the potent anti-tumor effect of cinnamon extract.

## Conclusions

Cinnamon extract potently inhibited various tumor cell growths *in vitro *and suppressed *in vivo *melanoma progression. Anti-cancer effect of cinnamon extract is mediated by apoptosis induction and blockade of NFκB and AP1. Hence, cinnamon extract could lead to development of potent anti-tumor agent or complementary and alternative medicines for the treatment of diverse cancers.

## Competing interests

The authors declare that they have no competing interests.

## Authors' contributions

HKK mainly performed this study. JSH, JSS, CGL, AS, JHR, WKJ and CRI helped some of experiments. BSK, SHL and ZYP contributed analytic tools. HKK and SHI designed the experiments and wrote the paper. All authors have read and approved the final manuscript.

## Pre-publication history

The pre-publication history for this paper can be accessed here:

http://www.biomedcentral.com/1471-2407/10/392/prepub

## Supplementary Material

Additional file 1 **Figure S1. Treatment of cinnamon extract induces cancer cell-specific apoptosis**. Tumor specific apoptotic effects of cinnamon by comparing induction of apoptotic population between normal mouse lymphocytes and B16F10 melanoma cells upon treatment of cinnamon extract or Doxorubicin. Click here for file

Additional file 2 **Table S1. Cinnamon treatment induced cell cycle alteration in tumor cells**. After treatment of cinnamon extract for indicated times (0, 48 and 72 hrs), cell cycle analysis of each sample was performed by propidium iodide staining. Click here for file

Additional file 3 **Figure S2. Treatment of cinnamon induces apoptosis in adenocarcinoma cell**. Effects of cinnamon extract treatment into Caco2 cells (human epithelial colorectal adenocarcinoma cell line) by checking alteration of apoptotic population. Click here for file

Additional file 4 **Figure S3. Oral administration of cinnamon extract induced cell death in tumor tissues**. *In vivo *tumor specific apoptosis by cinnamon treatment was confirmed by checking the structural changes of nucleus in tumor tissue. Click here for file
