# Correction to: Cinnamon extract induces tumor cell death through inhibition of NFκB and AP1

**DOI:** 10.1186/s12885-019-6342-5

**Published:** 2019-11-14

**Authors:** Ho-Keun Kwon, Ji-Sun Hwang, Jae-Seon So, Choong-Gu Lee, Anupama Sahoo, Jae-Ha Ryu, Won Kyung Jeon, Byoung Seob Ko, Sung Haeng Lee, Zee Yong Park, Sin-Hyeog Im

**Affiliations:** 1 School of Life Sciences and Immune Synapse Research Center, Gwangju Institute of Science and Technology (GIST), 1 Oryong-dong, Puk-ku, Gwangju, 500-712 Republic of Korea; 2 Korea Institute of Oriental Medicine, Daejeon, 305-811 Republic of Korea; 3 Chosun University School of Medicine, Gwangju, 501-759 Republic of Korea

**Correction to: BMC Cancer**

**https://doi.org/10.1186/1471-2407-10-392**

Following publication of the original article [1], the authors have re-evaluated the authorship for this article. The updated author group is:

Ho-Keun Kwon^1^ , Ji-Sun Hwang^1^ , Jae-Seon So^1^ , Choong-Gu Lee^1^ , Anupama Sahoo^1^ , Jae-Ha Ryu^1^ , Won Kyung Jeon^2^ , Byoung Seob Ko^2^ , Sung Haeng Lee^3^ , Zee Yong Park^1^ , Sin-Hyeog Im^1^*
