# Supplementary material for: Cinnamon extract induces tumor cell death through inhibition of NFκB and AP1
Source: BMC Cancer. 2010 Jul 24;10:392. doi: 10.1186/1471-2407-10-392 (PMC2920880; doi:10.1186/1471-2407-10-392)
Supplement: Additional file 1 — Figure S1. Treatment of cinnamon extract induces cancer cell-specific apoptosis. Tumor specific apoptotic effects of cinnamon by comparing induction of apoptotic population between normal mouse lymphocytes and B16F10 melanoma cells upon treatment of cinnamon extract or Doxorubicin. [file 1471-2407-10-392-S1.PDF]

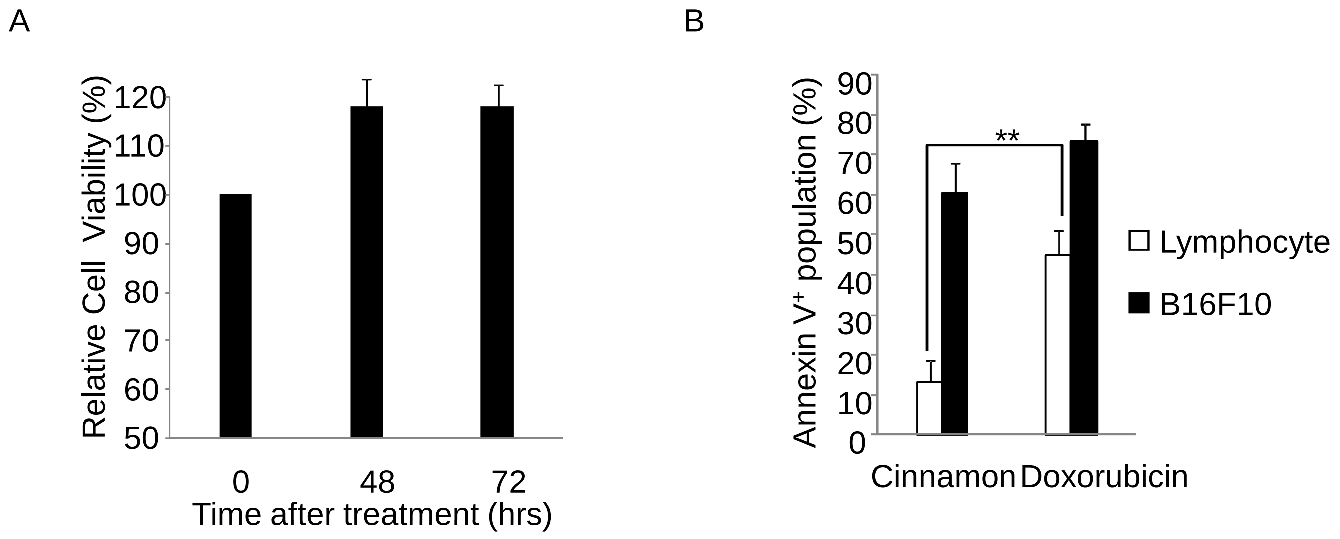

Figure S1. Treatment of cinnamon extract induces cancer cell-specific apoptosis.
(A) To test cinnamon effects in normal cell growth, cinnamon (0.5 mg/ml) was treated for 0, 48 and 72 hrs to mouse primary lymphocyte and then cell viability was measured at the indicated time points. (B) To compare anti-cancer effects of cinnamon extract (CE) with commercial anti-cancer drug, cinnamon (0.5 mg/ml) or Doxorubicin (Dox; 5 μM) was treated in B16F10 melanoma cells or normal mouse lymphocytes for 72 hrs and then Annexin V^+ population was measured by FACS. Error bars indicated SD. Data are representative of three independent experiments.
