# Supplementary material for: Cinnamon extract induces tumor cell death through inhibition of NFκB and AP1
Source: BMC Cancer. 2010 Jul 24;10:392. doi: 10.1186/1471-2407-10-392 (PMC2920880; doi:10.1186/1471-2407-10-392)
Supplement: Additional file 2 — Table S1. Cinnamon treatment induced cell cycle alteration in tumor cells. After treatment of cinnamon extract for indicated times (0, 48 and 72 hrs), cell cycle analysis of each sample was performed by propidium iodide staining. [file 1471-2407-10-392-S2.PDF]

Table S1

Cell cycle analysis				
	**Apoptotic**	**G0/G1**	**S**	**G2/M**
**0 hr**	1.92 ± 0.12	75.38 ± 0.53	10.84 ± 0.08	12.04 ± 0.32
**48 hrs**	5.84±0.23	70.36 ± 0.25	13.8 ± 0.04	10.15 ± 0.03
**72 hrs**	8.93±0.09	70.66 ± 0.39	12.23 ± 0.27	8.32 ± 0.10

Cinnamon treatment induced cell cycle alteration in tumor cells.
Description: After treatment of cinnamon for indicated times (0, 48 and 72 hrs), cell cycle analysis of each sample was performed by propidium iodide staining.
