# Supplementary material for: Cinnamon extract induces tumor cell death through inhibition of NFκB and AP1
Source: BMC Cancer. 2010 Jul 24;10:392. doi: 10.1186/1471-2407-10-392 (PMC2920880; doi:10.1186/1471-2407-10-392)
Supplement: Additional file 3 — Figure S2. Treatment of cinnamon induces apoptosis in adenocarcinoma cell. Effects of cinnamon extract treatment into Caco2 cells (human epithelial colorectal adenocarcinoma cell line) by checking alteration of apoptotic population. [file 1471-2407-10-392-S3.PDF]

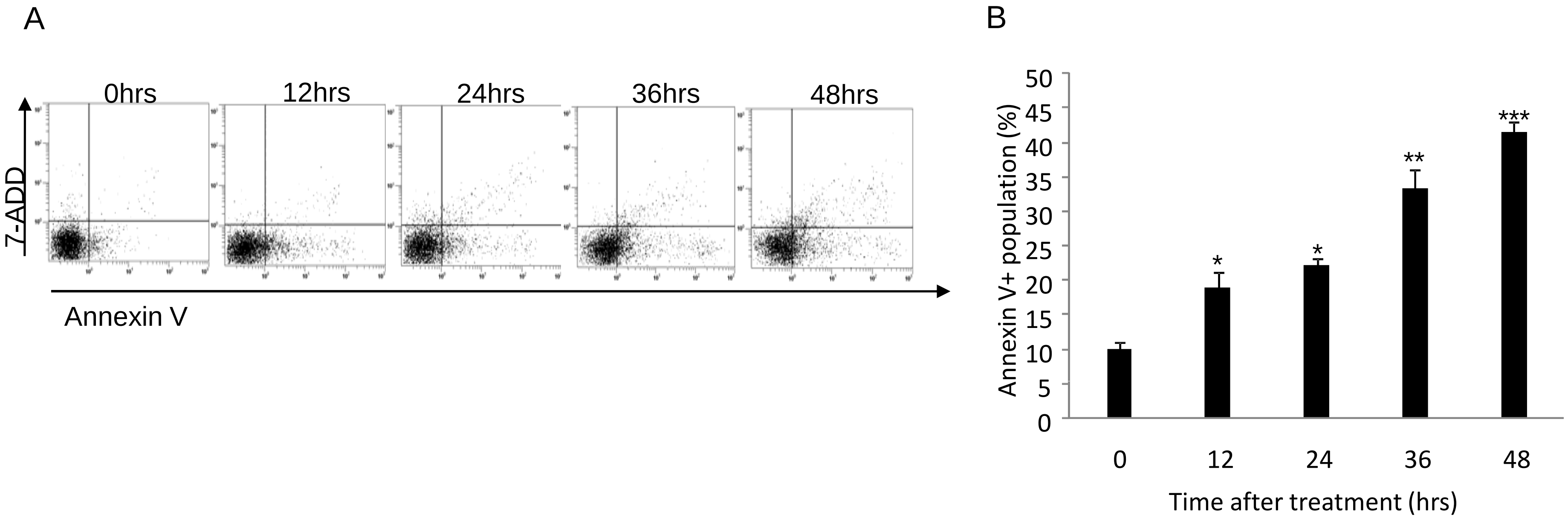

Figure S2. Treatment of cinnamon induces apoptosis in adenocarcinoma cell.
To test whether cinnamon extract could also induce apoptosis in other cancer cells, cinnamon extract (0.5 mg/ml) was treated in Caco2 cells (human epithelial colorectal adenocarcinoma cell line) for indicated time points. Apoptotic population was analyzed by staining with Annexin-V and 7-ADD at each time point. Error bars indicated SD. One (*), two (**) or three asterisks (***) indicate $p < 0.05$, $p < 0.005$ or $p < 0.001$, respectively. Data are representative of three independent experiments.
