# Supplementary material for: Cinnamon extract induces tumor cell death through inhibition of NFκB and AP1
Source: BMC Cancer. 2010 Jul 24;10:392. doi: 10.1186/1471-2407-10-392 (PMC2920880; doi:10.1186/1471-2407-10-392)
Supplement: Additional file 4 — Figure S3. Oral administration of cinnamon extract induced cell death in tumor tissues. In vivo tumor specific apoptosis by cinnamon treatment was confirmed by checking the structural changes of nucleus in tumor tissue. [file 1471-2407-10-392-S4.PDF]

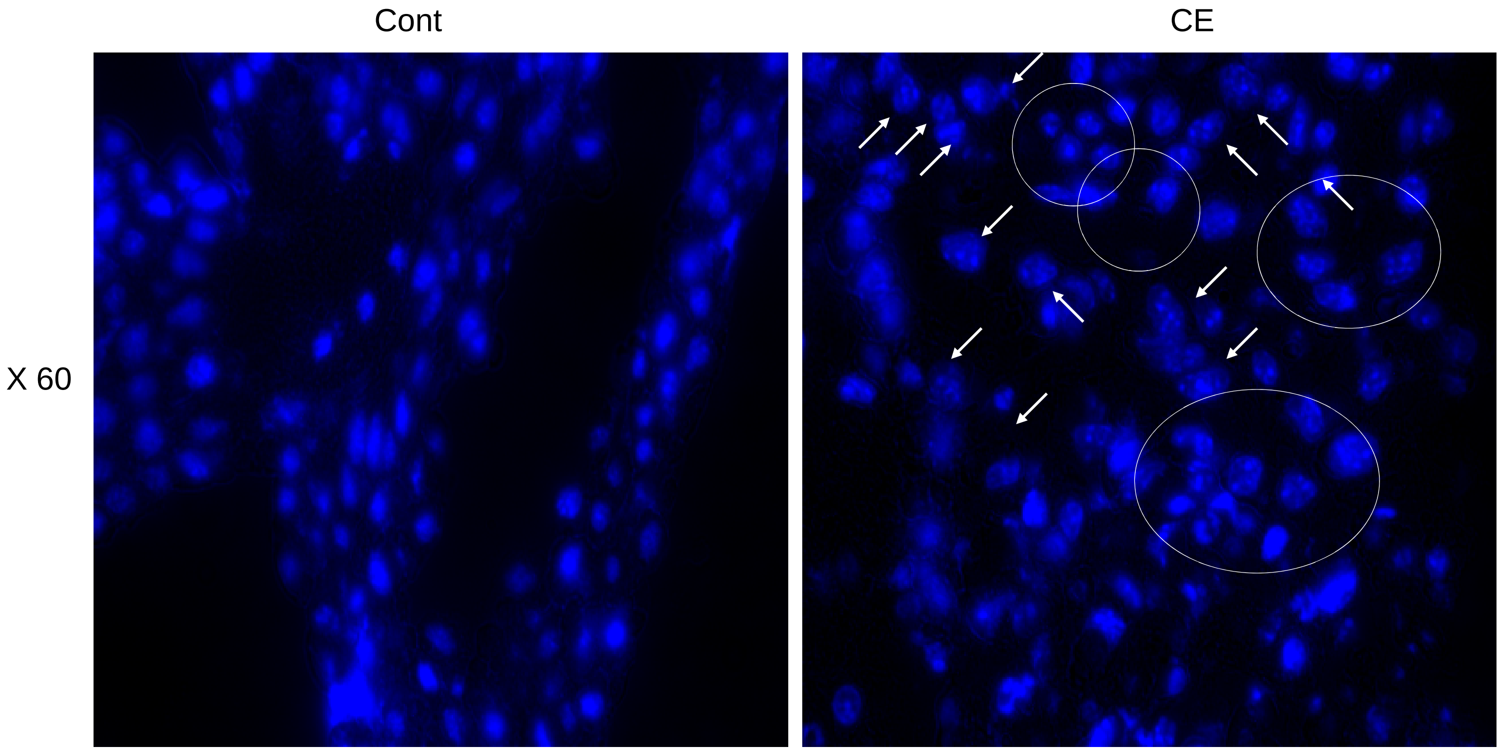

Figure S3. Oral administration of cinnamon induced cell death in tumor tissues.
To check the structural changes of nucleus, tumor tissues from each group (Cont; control, CE; cinnamon extract) were sectioned and then stained with Hoechst. Round areas and arrows indicate apoptotic cells.
